# Interferencia por macrocomplejos B12: hacia una detección eficaz e interpretación correcta de la hipo e hipervitaminemia

**DOI:** 10.1515/almed-2024-0126

**Published:** 2024-09-27

**Authors:** Jose Antonio Delgado, María I. Pastor, Gemma Costa, Nuria Márquez, Josep Miquel Bauça

**Affiliations:** Servicio de Medicina de Laboratorio, Hospital Universitari Son Espases, Palma, España; Institut d’Investigació Sanitària de les Illes Balears, Palma, España

**Keywords:** interferencia, intervalos de referencia, macromoléculas, cobalamina, biomarcador, B12

## Abstract

**Objetivos:**

Los resultados indicativos de hipervitaminemia B12 pueden deberse a la presencia de macro B12. Las biomoléculas de alto peso molecular se pueden eliminar mediante precipitación con polietilenglicol (PEG). Sin embargo, con esta técnica, se pueden coprecipitar moléculas “libres,” por lo que es necesario establecer nuevos valores de referencia para el resultado post precipitación. Los objetivos principales del presente estudio son: 1) Establecer intervalos de referencia (IR) post-PEG para la vitamina B12. 2) Comparar los dos criterios establecidos en la literatura para determinar la presencia de macro B12; 3) Evaluar la utilidad conjunta del porcentaje de recuperación de vitamina B12 post PEG y de los intervalos de referencia post PEG, para determinar el estado real de vitamina B12 en el organismo; y 4) Proponer comentarios para facilitar la interpretación de los resultados.

**Métodos:**

Se realizó un estudio prospectivo en el que se analizaron 488 muestras séricas de individuos “sanos” para determinar el porcentaje de recuperación y los intervalos de referencia tras la precipitación con PEG. Posteriormente, se realizó un análisis retrospectivo para evaluar la utilidad conjunta de ambas definiciones ante la sospecha de la presencia de macro B12. Se incluyó un total de 297 casos.

**Resultados:**

La recuperación y los IR post-PEG, determinados con la plataforma Alinity i oscilaron entre el 60 % y el 107 % y entre 118 y 506 pmol/L, respectivamente. La prueba de McNemar reveló diferencias estadísticamente significativas entre los dos criterios a la hora de estimar la interferencia. Sin embargo, ambas metodologías mostraron un elevado nivel de concordancia. En los 27 casos, la presencia de macro-B12 coincidió con hipervitaminemia de B12 confirmada. En ningún caso, la presencia de macro B2 coincidió con un déficit de B12.

**Conclusiones:**

Se debería incluir en los informes analíticos la concentración total de vitamina B12, el porcentaje de recuperación y la concentración de vitamina B12 post PEG, así como sus IR ajustados, con el fin de poder evaluar con mayor precisión el estado de la vitamina en el organismo.

## Introducción

La vitamina B12, también llamada cobalamina, es un micronutriente esencial que actúa como cofactor de la enzima metionina sintasa y la metilmalonil-CoA mutasa, que participan en la síntesis de ADN y los reordenamientos intramoleculares, así como en el neurodesarrollo [1].

A pesar de sus limitaciones, la cuantificación de la concentración sérica total de vitamina B12 es el biomarcador más ampliamente utilizado en la práctica clínica a la hora de evaluar el estado de la vitamina B12 y su metabolismo. A diario, los laboratorios clínicos reciben un elevado número de peticiones que incluyen la vitamina B12, ya que es una prueba económica y sencilla, que se realiza mediante inmunoensayo con analizadores automáticos de amplio espectro. El objetivo de dichas peticiones suele ser detectar un posible déficit de vitamina B12, ya que este está estrechamente relacionado con diversas patologías crónicas y los problemas asociados a las mismas, entre los que se encuentran la neuropatía y los trastornos cognitivos, la anemia, las patologías cardiovascular, la degeneración macular asociada a la edad y las enfermedades gastrointestinales [2, 3]. Sin embargo, una baja concentración sérica de B12 no siempre indica un déficit, de la misma manera que un valor dentro de los límites de normalidad no siempre implica normalidad [4, 5]. Así, los inmunoensayos para la determinación de vitamina B12 no están exentos de interferencias mediadas por anticuerpos, entre los que se incluyen el factor reumatoide, los anticuerpos heterófilos, los anticuerpos anti-factor intrínseco y los macrocomplejos de vitamina B12 [6, 7]. La detección de dichas interferencias supone todo un reto para el laboratorio clínico, ya que pueden enmascarar el estado real del paciente y derivar en acciones médicas inadecuadas. Esto adquiere especial relevancia en el caso de la vitamina B12, cuyo déficit y exceso están asociados a patologías potencialmente graves descritas en la literatura [5, 8], [9], [10.

La presencia de macrocomplejos de vitamina B12 (macro B12), habitualmente debida a la existencia de complejos de inmunoglobulina, puede derivar en un resultado analítico de hipervitaminemia de B12 (hiper B12), algo que muchos clínicos desconocen [5]. Se produce ante la presencia de moléculas circulantes de alto peso molecular, que son eliminadas lentamente de la circulación y no presentan actividad biológica, ni poseen ningún significado clínico conocido [8]. Cuando se detecta un caso de hiper B12, la detección temprana de una posible interferencia por macro B12 mediante el método de precipitación con polietilenglicol (PEG) llevará al clínico a plantearse la necesidad de realizar más pruebas médicas, ya que dicha interferencia puede enmascarar casos de déficit. Por otro lado, puede darse que la interferencia sea concurrente a una verdadera hiper B12.

A diferencia de otros biomarcadores como la prolactina, en la práctica diaria del laboratorio, rara vez se investiga la posible interferencia por macro B12 [11]. Además, no existe consenso sobre las situaciones en las que se debe investigar la presencia de macro B12 en los pacientes con hiper B12, ni sobre la manera de comunicar los resultados sobre la concentración de vitamina B12 post PEG. Esta falta de armonización puede afectar a la correcta interpretación de los resultados.

Un método sencillo para eliminar las biomoléculas de alto peso molecular en una muestra biológica es mediante precipitación con polietilenglicol (PEG). Sin embargo, este método presenta la limitación de que parte de la molécula “libre” puede coprecipitar. Este hecho invalida los intervalos de referencia que se emplean habitualmente para las muestras con presencia de dichas macromoléculas e impone la necesidad de establecer nuevos intervalos de referencia para el resultado post precipitación, dado que el porcentaje de recuperación no refleja si la concentración sérica del biomarcador se encuentra o no dentro de los límites de normalidad, por lo que resulta imposible evaluar el estado real de vitamina B12 de los pacientes. Algunos autores como Solemaini y col [12] se oponen a emplear el porcentaje de recuperación para determinar la presencia de macro B12 y definen la presencia de dicha interferencia cuando el valor absoluto del resultado de vitamina B12 total post PEG se encuentra por debajo de límite superior de referencia del intervalo de referencia post PEG (incluyendo el intervalo de confianza del 90 %).

Partiendo de estas observaciones, tal como ocurre en el caso de la prolactina, sería recomendable que se establecieran intervalos de referencia post PEG e incluirlos en los informes analíticos, junto con el porcentaje de precipitación, con el fin de garantizar la correcta interpretación del estado del biomarcador de interés [13].

Actualmente, en nuestro hospital aplicamos un protocolo anteriormente establecido para la detección de interferencias mediadas por anticuerpos en la prueba de vitamina B12 total. No obstante, hasta la fecha, no se dispone de intervalos de referencia post PEG.

Con el objeto de seguir mejorando, y siguiendo las recomendaciones de las sociedades científicas [14], el presente estudio tenía cuatro objetivos: 1) establecer intervalos de referencia post PEG (IR) para la vitamina B12 total en nuestra población a través de un estudio prospectivo; 2) comparar y evaluar la concordancia entre los dos criterios establecidos en la literatura en función de la presencia o ausencia de macro B12 mediante análisis retrospectivos; 3) evaluar la utilidad combinada de aplicar el porcentaje de recuperación de vitamina B12 post PEG y los IR post PEG para informar del verdadero estado de vitamina B12 (análisis retrospectivos), y en esta línea; 4) Proponer comentarios para facilitar la interpretación de los resultados.

## Materiales y métodos

Estos estudios observacionales se realizaron en el Hospital Universitari Son Espases (Palma de Mallorca, España), un hospital terciario que atiende a una población directa de 325.000 habitantes, siendo centro de referencia para una población de 1,200.000 habitantes, aproximadamente. La población es principalmente caucásica, con hábitos alimentarios correspondientes a la dieta mediterránea. El estudio prospectivo se realizó entre octubre de 2021 y junio de 2022, mientras que el análisis retrospectivo incluyó el periodo entre junio de 2022 y junio de 2023. Los datos analíticos fueron extraídos del sistema informático del laboratorio, GestLab (Indra, España). Por otro lado, la información clínica se extrajo del sistema informático del hospital, Millenium (Cerner Corporation, EE.UU), tras recibir la aprobación del Comité Ético de nuestra institución [*Comité de Ética de la Investigación de las Islas Baleares* (CEI-IB), nº IB 4775/22 PI].

### Sujetos

#### Estudio prospectivo: establecimiento de los IR post PEG para la vitamina B12 total

##### Criterios de inclusión

Se incluyó a todos los individuos ≥15 años con resultados dentro de los límites de normalidad para las siguientes magnitudes incluidas en una solicitud ordinaria: recuento de hematíes (hombres: 4,5–5,8 × 10^12^/L; mujeres: 3,8–5,4 × 10^12^/L), hemoglobina (hombres: 130–167 g/L; mujeres: 125–155 g/L), volumen corpuscular medio (80–96 fL), recuento de hematíes (4,0–11,0 × 10^9^/L), recuento de plaquetas (150–400 × 10^9^/L), alanina aminotransferasa (ALT) (<55 U/L), bilirrubina total (<20,5 µmoL/L), tasa de filtración glomerular estimada (CKD-EPI) (>90 mL/min/1,73 m^2^), proteína en orina de micción espontánea (tira de orina: <10^−1^ g/L), folato (7,0–46,5 nmoL/L), vitamina B12 (140–554 pmoL/L), y ácido metilmalónico en orina (MMA) (0,52–5,75 mmol MMA/mol creatinina).

Los intervalos de referencia de la vitamina B12 y del ácido metilmalónico en orina se determinaron previamente en nuestro laboratorio a través de dos estudios prospectivos.

##### Criterios de exclusión

Se revisó la historia clínica de los candidatos a inclusión, excluyéndose a aquellos que cumplieran los siguientes criterios: suplemento de vitamina B12 o folato; cualquier causa que provoque trastornos en el metabolismo de la vitamina B12 como el alcoholismo, patología hepática, enfermedad renal crónica (ERC), enfermedades autoinmunes, enfermedades infecciosas (VIH, hepatitis) y enfermedades hematológica; antecedentes de tumor sólido o enfermedad gastrointestinal (Crohn’s, malabsorción); enfermedades genéticas como la aciduria metilmalónica, y aquellos individuos con síntomas de neuropatía (entumecimiento y hormigueo en manos y pies, dificultades para caminar, etc.) o deterioro cognitivo (demencia, Alzheimer, etc.). Así mismo, se excluyó a aquellos individuos en tratamiento con inhibidores de la bomba de protones o metformina, embarazadas, e individuos con niveles séricos de vitamina B12 que presentaron interferencias mediadas por anticuerpos, determinadas en una plataforma Alinity i (Abbott Diagnostics, EE.UU) (macrocomplejos de vitamina B12, factor reumatoide, y anticuerpos heterófilos o antifactor intrínseco). También se excluyó a aquellos individuos cuya recuperación de vitamina B12 tras la precipitación con PEG fuera inferior al 60 % del valor original. Los puntos de corte para la confirmación o descarte de interferencia se obtuvieron de un estudio anterior [14].

#### Estudio retrospectivo: Utilidad de los criterios para definir la presencia o ausencia de macro B12 considerados tanto individualmente como combinados

##### Criterios de inclusión

**Figura 1:**
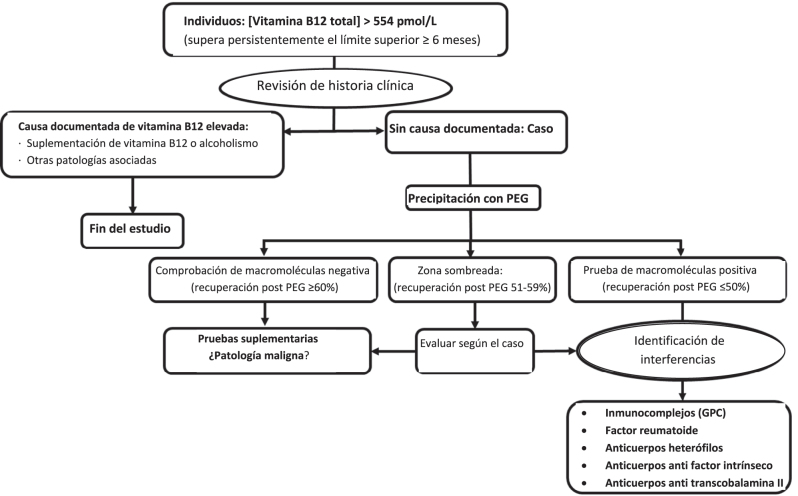
Algoritmo para la comprobación de interferencias debido a la presencia de macrocomplejos B12 en el laboratorio clínico.

Individuos con hiper B12 (>554 pmoL/L) que cumplieran los criterios de precipitación con PEG, según el algoritmo establecido en nuestro laboratorio (Figura 1).

##### Criterios de exclusión

Se excluyó a los individuos en los que se hubieran confirmado interferencias anteriormente en nuestra prueba de vitamina B12.

Con respecto al segundo objetivo de nuestro estudio, comparamos el valor diagnóstico de la definición de Solemaini y *col* (basada en la concentración de vitamina B12 post PEG) con la definición tradicional (basada en el porcentaje de recuperación de vitamina B 12) para determinar la presencia de macro B12.

Así mismo, con el objeto de evaluar la utilidad combinada de ambos criterios, los casos se clasificaron en función de los resultados combinados de porcentaje de recuperación y el resultado total de vitamina B12 post PEG. La presencia de interferencia mediada por anticuerpos (principalmente macro-B12) es *positiva* si la recuperación fue ≤50 % y *negativa* o *ausente* si es ≥60 %; estableciéndose una zona gris si la recuperación fue de entre el 51 y 59 %, cuando las muestras resultaron *dudosas*. Teniendo en cuenta los valores de vitamina B12 post PEG, el estado de hiper B12 se confirma cuando el valor total de vitamina B12 post PEG superaba el límite máximo del intervalo de referencia obtenido y propuesto por nuestro laboratorio. Del mismo modo, la hipovitaminemia se confirmaba si el valor total de vitamina B12 post PEG era menor que el límite inferior.

### Proceso analítico

La vitamina B12 se midió en suero mediante inmunoensayo de quimioluminiscencia en una plataforma Alinity i (Abbott Diagnostics, EE.UU). La calidad de la medición se garantizó mediante los procedimientos analíticos habituales, con un coeficiente de variación (CV) inferior al 6 % en el rango de concentraciones de los controles de calidad interna (269–674 pmol/L).

Todas las muestras séricas de los dos estudios se sometieron a precipitación con PEG, siguiendo el protocolo habitual [15]. Se mezclaron 200 µL de suero con 200 µL de una solución de PEG-6000 al 25 %. Se mezclaron las muestras y se centrifugaron a 2,200 g durante 15 minutos. A continuación, se midió la concentración de vitamina B12 en el sobrenadante. El porcentaje de recuperación de vitamina B12 tras la precipitación con PEG se calculó aplicando la siguiente fórmula:

$$\text{Recuperación}\;\left(\%\right)=\frac{2\times \left[B12\;\text{post}\;\text{PEG}\right]}{\left[B12\;\text{pre}\;\text{PEG}\right]}\times 100$$

### Análisis estadístico

La normalidad de la variable vitamina B12 post PEG se evaluó mediante el test de Kolmogorov-Smirnov. Dado que no seguía una distribución normal, el intervalo de referencia se determinó mediante cálculos no paramétricos (percentil 2,5-percentil 97,5). Las proporciones de muestras relacionadas se compararon mediante la prueba de McNemar. Aplicando el índice Kappa de Cohen, se evaluó la concordancia entre las dos definiciones (porcentaje de recuperación post PEG frente a la vitamina B12 total post PEG) a la hora de determinar la presencia o ausencia de macro B12.

La significación estadística se estableció en 0,05.

Los datos se procesaron en hojas Excel 2010 (Microsoft Inc, EE.UU) y MedCalc v.19.3 (MedCalc Software Ltd, Bélgica).

## Resultados

Para el estudio prospectivo, una vez aplicados los criterios de inclusión y exclusión, se incluyó a 488 individuos, 217 hombres y 271 mujeres con una edad media de 47 años (rango intercuartílico: 30–75), y una concentración de vitamina B12 sérica total de 328 pmol/L (rango intercuartílico: 251–399). El porcentaje de recuperación post PEG osciló entre el 60 % (IC_95%_: 59–61 %) y 107 % (CI_95%_: 106–108 %), con una media de recuperación del 90 %. Con respecto a los límites del intervalo de referencia para la vitamina B12 total post PEG calculados en función de los percentiles 2,5 y 97,5, los resultados fueron de 118 pmol/L (IC_95%_: 107–129) y 506 pmol/L (IC_95%_: 494–517), respectivamente.

Con respecto al análisis retrospectivo, 297 casos cumplieron los criterios de precipitación con PEG según el algoritmo establecido en nuestro laboratorio. Según la definición de macro-B12 basada en el porcentaje de recuperación (≤50 %), 105 casos (35 %) presentaron interferencia mediada por anticuerpos, mientras que diez casos fueron definidos como *dudosos* (3,4 %). Sin embargo, si se hubiera aplicado el criterio basado en el valor total de vitamina B12 post PEG propuesto por Solemaini y col, 102 casos (34 %) habrían presentado dicha interferencia. De los diez casos clasificados como *dudosos* siguiendo la definición basada en el porcentaje de recuperación post PEG, tres de ellos habrían sido clasificados como macro B12, según el criterio de Soilemaini.

En el análisis estadístico, solo se tuvieron los 287 casos donde se confirmó la *presencia* o *ausencia* de interferencia por macro-B12. La prueba de McNemar reveló diferencias estadísticamente significativas (valor p<0,001) entre los dos criterios a la hora de estimar la interferencia. Sin embrago, a pesar de la significación estadística, las dos metodologías mostraron un buen nivel de concordancia (Kappa corregido=0,65) en nuestro estudio.

En la Tabla 1 se muestra la clasificación de los 297 casos que se sometieron a precipitación con PEG, teniendo en cuenta el porcentaje de recuperación como criterio para determinar la presencia o ausencia de macro B12 y el valor total de vitamina B12 post PEG como criterio para determinar el estado real de vitamina B12 total. Cabe señalar que, en 27 casos, la presencia de macro B12 coincidió con hiper B12 real: recuperación media 27 % (IC_95%_: 24–31 %); media de vitamina B12 total post PEG 1024 pmol/L (IC_95%_: 776–1,270 pmol/L). En ningún caso, la presencia de macro B12 coincidió con un déficit de B12.

**Tabla 1:** Análisis retrospectivo de los casos en los que se realizó la precipitación con PEG.

		Clasificación (% de recuperación)		
		Ausencia de macro-B12 (≥60 %),	Macro-B12 (51–59 %) dudosa,	Presencia de macro-B12 (≤50 %),
		n (%)	n (%)	n (%)
Clasificación (post-PEG IR)	Hipovitaminemia B12 (<118 pmol/L)	0	0	0
	Normovitaminemia B12 (118–506 pmol/L)	21 (7,1 %)	3 (1,0 %)	78 (26,3 %)
	Hipervitaminemia B12 (>506 pmol/L)	161 (54,2 %)	7 (2,3 %)	27 (9,1 %)

Durante el periodo del estudio, se realizó una segunda precipitación con PEG en diez individuos, con un intervalo medio de 108 días. La diferencia media en el porcentaje de recuperación entre la segunda y la primera determinación fue del 7 %. En ningún caso, dicha diferencia derivó en un cambio en la clasificación del paciente (presencia o ausencia de macro B12). En siete de ellos, la presencia de hiper B12 sin macro B12 persistió, mientras que en tres pacientes ocurrió lo contrario.

En la Tabla 2 se muestran los comentarios interpretativos a incluir en el informe del laboratorio.

**Tabla 2:** Comentarios interpretativos sobre el informe de laboratorio para individuos con hipervitaminemia cuyas muestras fueron sometidas a precipitación con PEG.

		Porcentaje de recuperación post PEG			Comentario
		Ausencia de macro B12 (≥60 %)	Macro B12 dudosa (51–59 %)	Presencia de macro B12 (≤50 %)	
Valor de vitamina B12 post PEG	Hipovitaminemia B12 (<118 pmol/L)			X	Déficit de vitamina B12 tras la eliminación de interferencias debido a la presencia de macrocomplejos B12.
					Recomendamos realizar más pruebas para evaluar el déficit funcional de B12 y considerar suplementación de la vitamina.
	Normovitaminemia B12 (118–506 pmol/L)	X			Concentración normal de vitamina B12 en ausencia de macrocomplejos B12 normovitaminemia.
			X		Concentración normal de vitamina B12.
					El/la paciente presentó posibles interferencias debido a la presencia de macrocomplejos B12 (que no tienen ni actividad biológica ni relevancia clínica), que fueron eliminados.
				X	Concentración normal de vitamina B12.
					El/la paciente presentó posibles interferencias debido a la presencia de macrocomplejos B12 (que no tienen ni actividad biológica ni relevancia clínica), que fueron eliminados.
	Hipervitaminemia B12 (>506 pmol/L)	X			Hipervitaminemia B12 en ausencia de interferencias debido a la presencia de macrocomplejos B12.
					En caso de que no esté recibiendo suplementación, recomendamos realizar más pruebas.
			X		Hipervitaminemia B12 confirmada tras la eliminación de posibles interferencias debido a la presencia de macrocomplejos B12.
					En caso de que no esté recibiendo suplementación, recomendamos realizar más pruebas.
				X	Hipervitaminemia B12 confirmada tras la eliminación de posibles interferencias debido a la presencia de macrocomplejos B12.
					En caso de que no esté recibiendo suplementación, recomendamos realizar más pruebas.

## Discusión

Hasta donde sabemos, el presente es uno de los escasos estudios destinados a establecer un intervalo de referencia para la vitamina B12 post PEG y a llevar a cabo un análisis en profundidad de la utilidad de su inclusión en los informes de laboratorio.

Ante la progresiva automatización del laboratorio, la reducción de costes y la mejora en el acceso a los inmunoensayos, la vitamina B12 total se ha convertido en una prueba común en las revisiones médicas de rutina. A pesar de las continuas mejoras, se han identificado interferencias mediadas por anticuerpos en múltiples plataformas analíticas [12, 14, 15]. La precipitación con PEG es un método rápido, sencillo, económico y de amplio acceso para la detección de dichas interferencias. De hecho, este método ya se está aplicando con éxito en las pruebas de prolactina y tirotropina 12], [13], [14], [15], [16, aunque apenas se utiliza para la vitamina B12 total. A pesar de sus ventajas, la precipitación con PEG no es un método específico y su interpretación debe ser realizada con cautela.

Normalmente, se utiliza el porcentaje de recuperación del biomarcador como indicador de la presencia de interferencias por macroformas o anticuerpos que interfieren con el propio inmunoensayo. Sin embargo, este punto de corte carece de especifidad diagnóstica, dado que la presencia de interferencia puede coexistir con hipo o hipervitaminemia B12 [8], tal como se ha observado en otras moléculas como la prolactina [17, 18]. En dichas situaciones, la no disponibilidad de un intervalo de referencia post PEG puede dificultar la interpretación de los resultados y llevar al clínico a clasificar a los pacientes erróneamente. Esto ha impulsado a las sociedades científicas [13] a recomendar que se incluya la concentración de la forma “libre” del biomarcador en el informe del laboratorio, junto con los intervalos de referencia adecuados establecidos por el propio laboratorio siempre que sea posible.

En nuestro estudio, los resultados obtenidos para el intervalo y el porcentaje medio de recuperación post PEG concuerdan con los datos publicados en estudios anteriores [12, 19]. Sin embargo, con respecto a los intervalos de referencia, aun siendo similares a los descritos por Solemaini y col obtenidos con la plataforma Cobas 8000^®^ [12] (122,1–514,4 pmol/L), difieren de los propuestos por Öncel y col [19]. Estas diferencias podrían deberse al empleo de plataformas y pruebas diferentes para determinar la vitamina B12 total [Unicel DXI 800 (Beckman Coulter, EE.UU)], tamaños muestrales menores, y criterios de inclusión y exclusión distintos, sumado a la heterogeneidad en la metodología empleada para establecer los IR. Öncel y col comunicaron una modificación de los IR propuestos por el fabricante, basada en el porcentaje de precipitación obtenido en un grupo de control. Esta metodología podría no ser recomendable, ya que los valores propuestos por los fabricantes podrían no estar bien ajustados a la población de los estudios. Tal como ocurre con todos los analitos o magnitudes bioquímicas, los IR para la B12 post PEG se debería siempre establecer siguiendo las recomendaciones de CLSI [20].

Con respecto a la comparación de las metodologías empleadas para determinar la presencia de macro B12, el análisis estadístico reveló diferencias significativas entre los dos criterios. No obstante, se observó un buen nivel de concordancia. Solemaini y col [12] observaron una mejor aproximación del valor de vitamina B12 total post PEG, por lo que abogan por abandonar el porcentaje de recuperación como criterio diagnóstico para la presencia de interferencia por macro B12. La principal dificultad en el uso individual de esta metodología es la incapacidad de distinguir la hiper B12 real de una situación combinada (hiper B12 y macro B12). Desde nuestro punto de vista, el establecimiento de un intervalo de referencia post PEG es un complemento ideal al porcentaje de recuperación, debiendo ser incluido en los informes de laboratorio, en consonancia con la guía de consenso de la SEQC-SEEN para la prolactina [13]. Esta conclusión se ve respaldada por los resultados de nuestro análisis retrospectivo, donde el uso individual de cualquiera de las dos definiciones podría haber sido insuficiente a la hora de evaluar un caso de manera integral. Encontramos un claro ejemplo en los 27 casos (9,1 %) en los que, aparentemente, la presencia de macro B12 coincidía con la de hiper B12 (Tabla 1). Además, tal como se muestra en la Tabla 1, más de la mitad de los casos presentaron hiper B12 real sin la presencia de macro B12 (54.2 %), lo que viene a ser similar a lo observado por Öncel *y col* [19] (55,2 %). No comunicar la presencia de hiper B12 al clínico (a través del informe de laboratorio) puede implicar que no se sigan realizando pruebas médicas que podrían llevar al origen de dicha hiper B12.

Además, aunque en nuestro análisis retrospectivo no hallamos ningún caso real de hipovitaminemia durante el periodo del estudio, el empleo de IR post PEG también podría permitir su eficaz detección y comunicación. Aunque este fenómeno es menos común, su detección es igualmente esencial a la hora de evitar los efectos patológicos provocados por el déficit de vitamina B12.

En nuestro estudio, el 35 % de los casos presentaron una interferencia mediada por anticuerpos, lo que evidencia la elevada prevalencia de los macros B12 en pacientes seleccionados con hipervitaminemia. En el laboratorio clínico, rara vez se analiza la posible interferencia de macro B12, y existe un gran desconocimiento entre el personal médico, así como falta de experiencia sobre el tema, sumados a las dificultades a la hora de interpretar los resultados. La intención de nuestro grupo es dar visibilidad a este problema. De hecho, ya propusimos un algoritmo sencillo, económico y fácil de implementar en los laboratorios clínicos de todo el mundo para detectar la interferencia por macro B12. En el presente estudio, la intención era ir un paso más allá y realizar algunas recomendaciones sobre el informe de laboratorio con respecto a la comunicación de interferencias de macro B12 para individuos con hipervitaminemia que se someten a precipitación con PEG, tal como se muestra en la Tabla 2:

– En primer lugar, abogamos por incluir la concentración total de vitamina B12 en los informes de laboratorio, así como el porcentaje de recuperación y la concentración de vitamina B12 post PEG (supuestamente, B12 libre) con sus propios IR ajustados a la población atendida y derivada para someterse a una determinación de vitamina B12 total.
– También recomendamos incluir junto a los resultados del informe comentarios explicativos o interpretativos para facilitar al clínico la interpretación de los mismos y, paralelamente, para fines didácticos (Tabla 2).
– Finalmente, aunque es necesario realizar más estudios para investigar la persistencia de las macroformas B12, basándonos en nuestra experiencia y en la información disponible en la literatura relativa a las macroformas en otros biomarcadores [18], consideramos que los pacientes con hiper B12 en los que se haya descartado previamente la presencia de macro B12, no es necesario repetir el procedimiento de precipitación, lo que reduciría la carga de trabajo de los laboratorios de endocrinología. Por otro lado, recomendamos fehacientemente comunicar el valor de B12 post PEG (expresado en concentración) en las muestras de pacientes con interferencia conocida, ya que esto es crucial para el adecuado seguimiento de dichos pacientes.

La principal fortaleza de nuestro método para establecer IR es su naturaleza prospectiva, así como el tamaño muestral, el cumplimiento de las recomendaciones del CLSI para el establecimiento de IR, y la inclusión de múltiples filtros personalizados para la selección de individuos sanos. La revisión de historias médicas permite excluir a aquellos individuos con alguna circunstancia que podría afectar al metabolismo de la vitamina B12.

Con respecto a las limitaciones, la aplicación de una metodología pseudodirecta para establecer IR en los informes depende del sistema informático del laboratorio y del hospital. A pesar de haber realizado una selección meticulosa de individuos aparentemente sanos, estos no son verdaderamente individuos de referencia, lo que también representa una limitación a la hora de optimizar el establecimiento de IR. Con respecto al análisis retrospectivo, su propia naturaleza hace que la comparación de ambas definiciones para determinar la presencia de macro B12 solo sea una aproximación, debido a la imposibilidad de confirmar los casos mediante cromatografia.

## Conclusiones

En aquellos casos en los que se realice precipitación con PEG, los informes de laboratorio deberían incluir la concentración de vitamina B12 total, el porcentaje de recuperación, y la concentración de vitamina B12 post PEG, acompañados de sus propios IR ajustados a la población atendida, en consonancia con la guía de consenso de la SEQC-SEEN para la prolactina, ya que el uso individual de cualquiera de los dos criterios podría ser insuficiente a la hora de evaluar un caso de manera integral, tal como demuestra nuestro estudio. Esta información (casos de hiper B12), junto con sus correspondientes comentarios explicativos en el informe de laboratorio, permitiría la correcta interpretación de estado real de esta vitamina en el organismo, facilitando el diagnóstico y tratamiento precoz, mejorando así la seguridad del paciente y reduciendo la morbimortalidad.
